# Histone deacetylase activity is necessary for left-right patterning during vertebrate development

**DOI:** 10.1186/1471-213X-11-29

**Published:** 2011-05-20

**Authors:** Katia Carneiro, Claudia Donnet, Tomas Rejtar, Barry L Karger, Gustavo A Barisone, Elva Díaz, Sandhya Kortagere, Joan M Lemire, Michael Levin

**Affiliations:** 1 Department of Biology Center for Regenerative and Developmental Biology Tufts University 200 Boston Ave. Medford, MA 02155 USA; 2 Barnett Institute Northeastern University Boston, MA 02115 USA; 3 Department of Pharmacology School of Medicine University of California, Davis Davis, CA 95616 USA; 4 Department of Microbiology and Immunology Drexel University College of Medicine Philadelphia, PA 19129 USA

**Keywords:** *Xenopus*, left-right asymmetry, laterality, *Nodal*, HDAC

## Abstract

**Background:**

Consistent asymmetry of the left-right (LR) axis is a crucial aspect of vertebrate embryogenesis. Asymmetric gene expression of the TGFβ superfamily member *Nodal related 1 *(*Nr1) *in the left lateral mesoderm plate is a highly conserved step regulating the *situs *of the heart and viscera. In *Xenopus*, movement of maternal serotonin (5HT) through gap-junctional paths at cleavage stages dictates asymmetry upstream of *Nr1*. However, the mechanisms linking earlier biophysical asymmetries with this transcriptional control point are not known.

**Results:**

To understand how an early physiological gradient is transduced into a late, stable pattern of *Nr1 *expression we investigated epigenetic regulation during LR patterning. Embryos injected with mRNA encoding a dominant-negative of Histone Deacetylase (HDAC) lacked *Nr1 *expression and exhibited randomized sidedness of the heart and viscera (heterotaxia) at stage 45. Timing analysis using pharmacological blockade of HDACs implicated cleavage stages as the active period. Inhibition during these early stages was correlated with an absence of *Nr1 *expression at stage 21, high levels of heterotaxia at stage 45, and the deposition of the epigenetic marker H3K4me2 on the *Nr1 *gene. To link the epigenetic machinery to the 5HT signaling pathway, we performed a high-throughput proteomic screen for novel cytoplasmic 5HT partners associated with the epigenetic machinery. The data identified the known HDAC partner protein Mad3 as a 5HT-binding regulator. While Mad3 overexpression led to an absence of *Nr1 *transcription and randomized the LR axis, a mutant form of Mad3 lacking 5HT binding sites was not able to induce heterotaxia, showing that Mad3's biological activity is dependent on 5HT binding.

**Conclusion:**

HDAC activity is a new LR determinant controlling the epigenetic state of *Nr1 *from early developmental stages. The HDAC binding partner Mad3 may be a new serotonin-dependent regulator of asymmetry linking early physiological asymmetries to stable changes in gene expression during organogenesis.

## Background

Despite a bilaterally-symmetrical bodyplan, many animals exhibit a consistent asymmetry in the placement and shape of the heart, viscera, and brain [1]. The wide-spread conservation of laterality, and the consistent linkage of the orientation of the left-right (LR) axis with the dorso-ventral and anterior-posterior axes (in a world that does not distinguish left from right above the quantum level), make LR patterning a fascinating problem [2-4]. In addition to its relevance to basic cell, developmental, and evolutionary biology, laterality presents significant implications for normal physiology and a plethora of clinically-important human syndromes [5,6]. Errors in LR patterning include loss of asymmetry (isomerism), complete inversions (*situs inversus*), and random placement of individual organs (loss of concordance known as heterotaxia).

It is widely accepted that large-scale LR asymmetry derives from the molecular chirality of subcellular structures [7]. However, at least two main classes of models have been proposed for how this chirality is propagated, amplified, and imposed on multicellular fields during development. One popular model focuses on the net unidirectional extracellular fluid flow achieved during gastrulation by the movement of cilia [8,9].

A different model focuses on much earlier stages, prior to gastrulation, when physiological events leverage asymmetry from the chirality of the intracellular cytoskeleton to set up asymmetrical movement of morphogens through cell fields [10-12]. One such morphogen is serotonin (5HT): a neurotransmitter of clinical relevance that has interesting roles outside the central nervous system [13]. In two vertebrate species (chick and frog), serotonergic signaling has been shown to be required for LR patterning. In the frog embryo, it is known that 5HT accumulates in the right blastomeres in a rapid process dependent on asymmetric voltage gradients across the midline and the presence of open gap junctions through which it traverses [14-16].

In *Xenopus *embryos, many aspects of this system have been elucidated: the source of the electrophoretic force driving this 5HT gradient has been molecularly characterized [14,17,18], and indeed many aspects are known in enough quantitative detail to allow the whole system to be computationally modeled [19,20]. However, one fundamental question has not been addressed: how does this physiological gradient, occurring in the frog at a time when the zygotic genome is mostly quiescent, couple to the later transcriptional cascade of asymmetrically expressed genes that is known to control organ positioning? Specifically: how does the arrival of 5HT within the right-side blastomeres control gene expression? Well-known 5HT receptor families [21] are not ideal candidates because they are functional on the outside surface of the plasma membrane, while the 5HT arrives through gap junctions, and thus requires an intracellular binding target.

To better understand how intracellular 5HT signaling is transduced into stable gene expression, from early to very late developmental stages, we hypothesized the involvement of epigenetic machinery as a new component of the LR establishment. Such a mechanism is attractive because once epigenetic markers are deposited on the chromatin, they remain stable along successive cell divisions carrying the epigenetic signature of an activated or repressed state of the chromatin. More specifically, we sought to determine whether early manipulation of the epigenetic state of the embryo would affect LR-relevant genes expressed at late developmental stages (e.g., *Nr1*).

Changes in the state of the chromatin have a key role for the proper control of gene expression during development. Post-translational modifications such as lysine acetylation constitute a code allowing specific interactions between chromatin and DNA binding proteins that ultimately will dictate the status of activation of a gene [22]. These modifications take place at the chromatin level and involve the acetylation of lysines in the amino terminal tail of core histones [23].

HDACs are important players for epigenetic memory control as they act by decreasing the levels of acetylated histones leading to chromatin compaction and repression [24,25]. HDACs play a critical role in regulating gene expression and aberrant levels of protein acetylation have been associated with cancer [26] and a range of neurological diseases such as fragile X mental retardation [27]. Indeed, HDACs blockers can reverse silent heterochromatin to an active conformational structure leading to the normal function of genes that are silent in these pathologies [27]. Despite their prominent role on gene expression, HDACs can not bind to the DNA on their own, but are recruited to specific points on the chromatin via interaction with transcriptional repressive complexes such as those containing Mad proteins [28].

Vertebrate HDACs can be classified into four groups: Class I (HDAC 1,2,3,8), Class IIa (HDAC 4,5,7,9) Class IIb (HDAC 6) and Class IV (HDAC 10,11). *Xenopus *has a maternal form of HDAC (HDAC) that shows sequence homology to other HDAC [29]. The HDAC activity in early embryos appears to be mainly, if not exclusively, of the HDAC-A type, that is of the vertebrate HDAC I class [30]. Although HDAC function during *Xenopus *development remains unclear, several studies have demonstrated its importance for vertebrate development. Genetic deletion of HDACs in mice and embryonic stem cells results in problems in proliferation [31,32], while the deletion of *hdac1 *in zebrafish leads to defects in skeletal and neuronal development [33,34]. Interestingly, in HDAC-null ES cells, only 3% of genes are downregulated and about 5% are upregulated. This suggests that the epigenetic machinery is directed to specific points of the chromatin, and indicates that HDACs could underlie regulation of specific developmental signals [35].

Here, we probe the involvement of HDACs in LR asymmetry establishment. Using several molecular and pharmacological tools, we found that HDAC activity is a new component of LR patterning that acts very early in embryonic development (prior to active zygotic transcription). In addition, we identified the HDAC-interacting protein Mad3 as a new component during LR development in the 5HT signaling pathway. Our results demonstrate how very early physiological signaling can be transduced into a stable pattern of gene expression at late developmental stages, and sheds light on how epigenetic state and chromatin structure orchestrate important events during early stages of axial patterning in animal development.

## Methods

### Constructs

Plasmids containing *X. laevis *Mad3 and *X. laevis *maternal HDAC cDNA were purchased from Open Biosystems (Clone ID: 4175511 and Clone ID: 6862376, respectively) and the DN HDAC was generated by PCR as described [36]. pCS2Vp16 and pCS2Eng were provided by Dr. D. Kessler and the constructs pCS2EngMad3 and pCS2Vp16Mad3 were generated by PCR cloning. To induce ectopic expression of *Xenopus *Mad3 and HDAC, their coding regions were inserted into the pCS2-flag expression vector, resulting in the addition of the flag-tag to the N-terminus of the coding region of Mad3. Mad3 and HDAC pCS2 plasmids were linearized with NotI to prepare for transcription with the SP6 Message machine kit (Ambion). Mad3-5 mut (Gln125 and Gln161 were replaced by glutamate; Asp145, Asp148 and Asp163 were replaced by asparagine) was generated by PCR mutagenesis with an Agilent kit.

### *Xenopus *embryos microinjection

For microinjections, capped, synthetic mRNAs [37], generated using the Ambion mMessage mMachine kit were dissolved in water plus the lineage tracer rhodamine-labeled dextran (RLD) and injected into embryos in 3% Ficoll. 30 minutes post-injection, embryos were transferred to 0.1X MMR and allowed to develop until stage 21 for *in situ *hybridization or stage 45 for organ placement score. All experimental procedures involving the use of animals for experimental purposes were approved by the Institutional Animal Care and Use Committees (IACUC) and Tufts University Department of Lab Animal Medicine (DLAM) under the protocol number M2008-08.

### Scoring for Organ *situs*

At stage 45, embryos were anesthetized with 5% tricaine and analyzed for position (*situs*) of 3 organs: the heart, stomach, and gallbladder [38]. Heterotaxia was defined as reversal in position of one or more organs. Only embryos with normal dorsoanterior development (DAI = 5) were scored to avoid scoring instances of secondary randomization due to errors in the dorso-ventral (DV) or antero-posterior (AP) axial patterning [39], and only clear left- or right-sided organs were scored. Percent heterotaxia was calculated as the number with heterotaxia divided by the number of total scorable embryos, i.e. embryos normal in all other ways. A χ^2 ^test was used for further statistical analysis.

### Whole amount *in situ *hybridization

Embryos were collected at different stages of development and fixed in MEMFA for 3 hours at room temperature and used for *in situ *hybridization as described in Harland (1991). Plasmids containing *Xenopus *Mad3 and *Xenopus *HDAC cDNA were purchased from Open Biosystems (Mad3 clone ID: 4175511; HDAC clone ID: 6862376) and cloned in pCS2. The plasmids were linearized and anti-sense probes for *in situ *hybridization were generated *in vitro *using DIG labeling mix from Invitrogen.

### *Xenopus *embryo drug treatment

Batches of embryos were separated into experimental and control groups and exposed to 0.1X MMR (control) or 0.1X MMR containing 100 mM Sodium Butyrate (NaB) (Sigma) during different stages of development. The drug was washed out and the embryos were allowed to develop until stage 21 for Nr1 *in situ *or until stage 45 for organ placement score.

### Western Blotting and Coimmunoprecipitation (CoIP) assay

For Western blottings embryos were collected at stage 7 (64 cells) and homogenized in lysis buffer (100mM NaCl, 20 mMNaF, 50 mM Tris pH 7.5, 5 mM EDTA, 1%NP40, 1% deoxycholate, 1:50 EDTA-free Complete protease inhibitor, Roche). The lysate was centrifuged for 15 minutes at 4°C and the supernatant was collected and frozen. The extracted proteins were subjected to SDS-PAGE and blotted onto a PVDF membrane (BioRad). After blocking with 5% skim milk and 0.1% Tween 20 in PBS, membrane filters were incubated with an anti-Mad3 (NeuroMab clone N129/15.1, supernatant) or anti-acetyl H4 (Milipore 1:500) overnight at 4°C. The membranes were washed in PBT and incubated with secondary HRP conjugated antibody (1:1000, Jackson). Immunosignals were visualized with chemoluminiscence.

For Co-IP assays, embryos were injected at the 1 cell stage with Mad3WT-flag or Mad3-5mut-flag constructs and collected at stage 7 (64 cells) when 10 embryos were homogenized in lysis buffer. A total of 100 μl of embryo lysate was incubated with 2 μg anti-5HT (AB125, Milipore) or rabbit IgG for 3 hours at 4°C followed by incubation with proteinA-agarose for 1 hour at the same temperature. The beads were collected by centrifugation and washed in lysis buffer. The samples were then washed in low salt buffer (50 mM Tris HCl pH 7.5, 0.1%NP40, 0.05% Deoxycholate) and high salt solution (50 mM Tris HCl pH 7.5, 500 mM NaCl, 0.1% NP40, 0.05% Deoxycholate). Proteins were eluted by adding SDS-PAGE sample buffer followed by boiling for 5 minutes. For Western blotting embryo lysates were loaded on 4-12% gradient polyacrylamide gel (Invitrogen) in sample buffer and the proteins were transferred to a nitrocellulose membrane, blocked in 5% non- fat milk in PBT (PBS plus 0.1% Tween 20) for 1 hour at room temperature and incubated overnight with anti-flag (Sigma; 1:1000). Membranes were washed with PBT and incubated with secondary antibody conjugate to HRP. The blotting was developed with a luminescence kit (Pierce) and the images were acquired with a camera coupled to a GBox system (Syngene).

To determine whether NaB treatment was effective, whole embryo lysates were prepared from embryos exposed to NaB at stages 1-7, stages 7-8, and stages 8-9, including also untreated stage-matched controls. The whole lysate from treated and control groups were prepared in the lysis buffer as described above and boiled for 5 minutes. The proteins were separated by SDS-PAGE and transferred to nitrocelulose membranes. Blots were then incubated with anti-acetyl H4 (Milipore) and developed with chemoluminiscence. To determine the relative abundance of acetylated histone H4, membranes were stripped of antibodies in 200 mM glycine buffer pH 2.2 and re-probed with anti-Tubulinα (Sigma, 1:4000). For quantification of acetylated histone H4 protein, the luminosity of individual bands was defined using the histogram function of Photoshop and normalized against Tubulin. Only exposures in the linear phase of detection were used for quantification. SEM was defined from normalized acetylated histone H4 and measured in three independent experiments. Statistical analysis was performed with Student's t-test.

### Chromatin preparation for Chromatin Immunoprecipitation (ChIP) experiments

Embryos were exposed to 100 mM NaB from stage 1 to 7, when the drug was washed out and the embryos were allowed to develop in 0.1X MMR until stage 21. The chromatin was then collected and the ChIP was performed following the protocol [40]. The purified chromatin from treated and control groups was then incubated with anti-acetyl H4, anti-acetyl H3, anti-H3K4me2 (Milipore) or anti-rabbit IgG (Milipore) as control. Amplification of the precipitated DNA was carried out in an Applied Biosystems Step One Plus PCR machine, using the standard SYBR green program with an initial melt stage at 95°C for 10 min, followed by 40 cycles of 95°C for 15 sec and 60°C for 1 min. The run was finished by a melt curve from 95°C to 60°C to ensure that no primer dimer artifacts were formed and that cycle threshold (Ct) values represent the desired amplicon. Primer sequences were designed with Primer 3 plus program (http://www.bioinformatics.nl/cgi-bin/primer3plus/primer3plus.cgi).

qPCR primers set used: *Nr1 *intronic region: Forward 5' - TTCCCTATTGACAGGGGTTG - 3'; Reverse 5'- GCCAAATGTCAAAACACTCG -3'. *Nr1 *Promoter region: Forward 5' - TCCTTGATGAGGCCATTAGC - 3': Reverse 5' - CAAACAGAGCATTCCCTGAC - 3'. The ΔΔCt method was used to represent the data as in [41] and each experiment was performed in triplicate and combined for further statistical analysis.

### Chromatographic 5HT affinity capture screening

One gram of Sepharose 4BCL powder was activated according manufacturer's instructions, washed and equilibrated in 4 mL buffer (50 mM TrisHCl, pH 7.5, 100 mM NaCl) plus 5 mM sodium metabisulfite to conjugate of 5-HT to the resin. Metabisulfite was added to avoid rapid 5HT oxidation at the alkaline pH necessary for the reaction. The 5HT capture was performed in batch and metabisulfite 5 mM was added throughout the whole process. Two 1 mL aliquots of conjugated resin were centrifuged and then loaded with 1 mL of 1 cell embryo lysate prepared in lysis buffer (0.5% SDS; 150 mM NaCl; 5 mM EDTA; 10 mM Tris, pH. 7.6; 2mM PMSF at a final protein concentration of 15 mg/mL) plus 20 mM 5HT (negative control) or 1 mL of 1 cell embryo lysate. The mixtures were incubated overnight at 4 C, after which the resins were washed three times at room temperature for one hour each with 50 mM TrisHCl pH 7.5, 100 mM NaCl at 4C to remove loosely bound proteins. Elution was performed in both cases by rotating the resin for one hour at room temperature with the same buffer plus 20 mM 5HT. The eluates were concentrated by centrifugational filtration with a cutoff of 3 kDa. The proteins from each fraction were subjected to a 10% SDS-PAGE and, after sample preparation and trypsin in-solution digestion, the peptide mixtures from the eluates were analyzed by LC/MS-MS using a Dionex 3000 LC (Sunnyvale, CA) coupled to a linear ion trap mass spectrometer (LTQ, Thermo Fisher, San Jose CA). The raw data collected on the LTQ was analyzed with Sequest software (Thermo Fisher) for protein identification using a database for *Xenopus laevis*.

### Mad3 modeling and simulation methods

The protein sequence of *Xenopus *Mad3 transcription factor (Q0VH33) was obtained from Swiss-Prot repository (http://www.expasy.ch/sprot). To identify a suitable template for modeling the 3D structure, the sequence was queried against the protein databank (PDB) sequences using Blast search engine [42]. Based on the results from these analysis, the crystal structure of lipocalin AM182, which is a paralog of monotonin, in complex with 5HT [43] was used as a template to model the non-DNA binding region of Mad3, using the homology modeling program MODELLER (ver 9.4, [44]. Ten models were built and ranked using the Modeller's objective function. The best ranking structure was subjected to 5000 energy minimization steps to relieve steric and geometric strains using NAMD (Ver.2) with charmm force field parameters. The 3D structure of 5HT was obtained from the co-crystal structure of AM182, hydrogen atoms were added and the structure was minimized using Amber charges and Amber force field as adopted in MOE program (Ver.2008.10, http://www.chemcomp.com). In the lipocalin structure, 5HT has salt bridge with Asp106 and Ser18 [43]. Among class-A GPCRs (G protein-coupled receptors), all biogenic amines including 5HT have a conserved salt bridge interaction with either an aspartic acid or glutamic acid residue in the third transmembrane region that is required for agonist and antagonist activity of GPCR ligands [45]. Based on this evidence, the proposed site for docking 5HT in Mad3 structure was identified and consisted of Asp163, Asp145, Asp148, Gln125, Gln161. 5HT was docked to the proposed binding site using the docking software Gold (v4.1) [46]. Twenty independent runs were performed to completely sample the ligand conformation and to avoid local minima and all the docked complexes were scored using Goldscore [46] and chemscore [47]. The best ranking complex was minimized using MOE as described above.

### Mad3 and 5HT in vitro interaction assay

#### Recombinant proteins

For use in binding experiments, Mad3 protein was obtained as a fusion protein with glutathione-S-transferase (GST) by transgenic expression in *Escherichia coli *and subsequent purification using glutathione (GSH)-agarose affinity chromatography. Briefly, the full Mad3 coding sequence was amplified by PCR using previously cloned Mad3 cDNA from human cell lines as a template. PCR reactions were performed under standard conditions using *Pfx *DNA polymerase (Invitrogen). PCR products were cloned into pCR-Blunt II-TOPO (Invitrogen) and subsequently subcloned into pGEX-5X-1 (GE Healthcare) using BamHI/SalI sites introduced by the PCR primers. All constructs were confirmed by sequencing. To express GST-Mad3, transformed E. coli BL21 were grown overnight at 37°C, diluted 200-fold in fresh medium and grown at room temperature until OD_600 _= 0.5-0.8. The culture was induced with 0.1 mM IPTG for 3 hours at room temperature. To express GST, E. coli BL21 were transformed with the empty vector, grown at 37°C to OD_600 _= 0.5-0.8 and induced with 1 mM IPTG for 1 hour at 37°C. After induction, pelleted cells (6,000 × *g*, 20 minutes, 4°C) were resuspended in 5 ml BugBuster protein extraction reagent (EMD Biosciences) per gram of wet cell paste and lysed with 100 μg/ml each lysozyme and DNAse I for 20 minutes at room temperature. Lysates were cleared by centrifugation for 20 minutes at 16,000 *g *and the supernatants were applied directly to a GSH-agarose column. Proteins were purified using standard procedures. Bound proteins were eluted with 25 mM GSH/50 mM Tris pH 8. The eluates were concentrated and the buffer changed to HBS-EP (10 mM Hepes pH 7.4, 150 mM NaCl, 3 mM EDTA, 0.005% surfactant P20) using an Amicon Ultra-15 device. Typically, GST was expressed in large quantities in the soluble fraction, while GST-Mad3 was found mostly in inclusion bodies, with a small percentage in the soluble fraction from which it was purified. Protein concentration was determined by the BCA method and the purity of the fractions was confirmed by SDS-PAGE.

#### Surface Plasmon Resonance

Binding experiments were carried out on a Biacore X system (GE Healthcare). Proteins of interest were immobilized on a CM5 chip using the Amine Coupling Kit (Biacore, GE Healthcare). Specifically, two cells on a CM5 chip were activated using a 1:1 ratio of N-hydroxysuccinimide (NHS):1-ethyl-3-(3-dimethylaminopropyl) carbodiimide (EDC) at a flow rate of 5 μl/min for 10 min. Recombinant GST and GST-Mad3 proteins were prepared as described above at a final concentration of 50 μg/ml and 10 μg/ml respectively in HBS-EP buffer (GE Healthcare). Prior to injection, 40 μL of protein solution were mixed with 30 μL of 100 mM Glycine pH 2.5, since these were the optimum conditions for capture as determined by pre-concentration experiments. GST was immobilized in the reference cell Fc2 by one 10 μL-injection at 10 μL/min. GST-Mad3 was immobilized in the sample cell Fc1 by five 20 μL-injections at 10 μL/min. Both Fc1 and Fc2 were subsequently blocked with 1 M ethanolamine (pH 8.5) for 7 min at 10 μl/min. A total of 7,962 response units of GST and 6,938 response units of GST-Mad3 were immobilized. Binding of 5HT was carried out at 25°C at 160 μL/min in HBS-EP. The amount of specific analyte bound was monitored by subtracting the response units from the reference cell (GST) from the GST-Mad3 immobilized cell. After each analyte injection, surfaces were regenerated with 50 mM NaOH for 0.25 sec at 160 μL/min. All sensorgram data presented are representative of at least 3 runs. Sensorgrams were aligned to the injection time (defined as t = 0 s) using BiaEvaluation software, and the baselines averaged at response difference = 0 RU. Curve fitting and binding kinetics parameters were calculated using GraphPad Prism Software.

## Results

### The epigenetic machinery controlled by HDAC is involved in LR patterning upstream of *Nodal related 1 *expression

**Figure 1:**
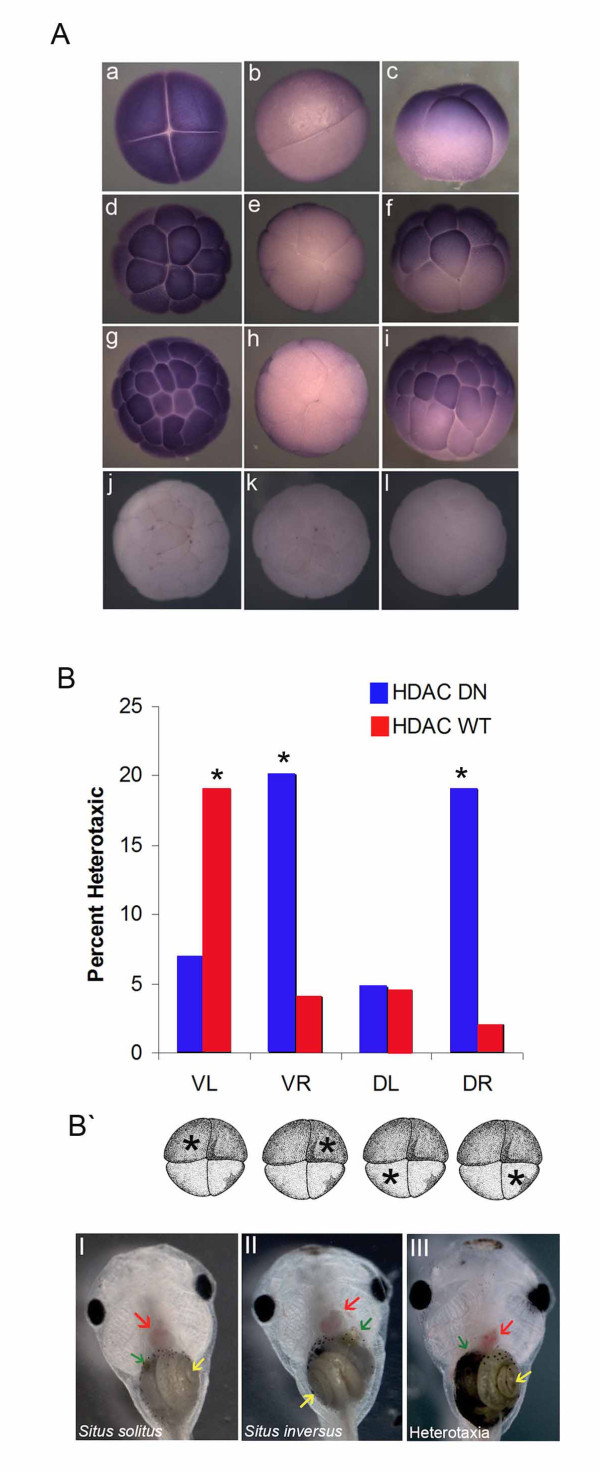
**Early HDAC mRNA injection induces heterotaxia**. (A) *Xenopus *HDAC mRNA expression pattern is shown by *in situ *hybridization with early *Xenopus *embryos. HDAC mRNA presents a symmetric expression pattern by animal cells at the stage 3 (4 cells) (a,b,c), at the stage 5 (16 cells) (d,e,f) and at the stage 7 (64 cells) (g,h,i). *in situ *reaction control with no secondary (j, animal view) or no probe added to the reaction (k, animal view; l, vegetal view). (B) Embryos injected with HDAC DN on the ventral or dorsal right blastomeres presented significant levels of heterotaxia (VR 20% heterotaxia *p *< 0.01, n = 55; DR 19% heteroatxia *p *< 0.01, n = 42; VL 7% heterotaxia *p *= 0.3 n = 55; DL 2% heterotaxia *p *= 0.7). Embryos injected with HDAC WT on the ventral left side presented significant levels of heteroatxia (VL 19% heterotaxia *p *< 0.01, n = 68; VR 4% heterotaxia *p *= 0.8, n = 49; DL 4% heteroataxia *p *= 0.8, n = 44; DR 2% heterotaxia *p *= 0.5, n = 50). (B`) Schematic showing the injected blastomere at the 4 cell stage. (I) Wild type phenotype showing the gut coil to left (yellow arrow), the gall bladder on the right (green arrow) and the heart loop on right (red line). (II) *Situs inversus *phenotype where all three organs analyzed are found inverted. (III) Heterotaxic phenotype showing the heart loop to the left (red arrow).

*Xenopus *embryos express a maternal form of HDAC mRNA that shows sequence homology to other HDACs [29]. The HDAC activity present in early embryos appears to be mainly, if not exclusively, of the HDAC-A type, that is of the vertebrate HDAC I class [30]. Although the biochemical properties of the *Xenopus *HDAC are well characterized [30], knowledge about its mRNA expression pattern is limited. We processed different stages of early embryos for *in situ *hybridization. HDAC mRNA was found to be expressed in all animal-pole blastomeres by the 64 cell stage (stage 7) (Figure 1A).

To probe the overall potential involvement of HDACs during *Xenopus *LR patterning, we injected early embryos with mRNA encoding a well-characterized dominant negative (DN) form of the *Xenopus *HDAC [36]. Embryos in which the DN HDAC mRNA was injected at the 1 cell stage exhibited a 17-fold increase in heterotaxia compared to controls (17% vs. 1% heterotaxia in control non injected embryos, n = 62, significant at *p *= 0.002), demonstrating that HDAC is indeed functionally involved in embryonic LR patterning in *Xenopus*. It should be noted that the penetrance of the phenotype is artificially reduced (compared to those reported in mouse models, which often have midline or other defects) in these experiments by titering down the reagents to sub-optimal levels (because of our stringent requirement of normal dorso-anterior index and purity of LR phenotype). Nevertheless, given the very low background incidence of heterotaxia in un-manipulated embryos, the level of laterality disturbance induced by our manipulations is unmistakably significant. Moreover, when scoring three organs for *situs*, the maximum level of heterotaxia is 87.5%, not 100%, since random independent assortment of 3 organs will sometimes result in a wild-type phenotype being mistakenly scored when all three organs randomly land in their correct positions.

Next we sought to investigate whether the left and right sides of the embryo require different levels of HDAC activity for proper organ *situs*. To test this hypothesis, we injected embryos at the 4 cell stage on the left (ventral or dorsal) or on the right (ventral or dorsal) side with the constructs HDAC DN or HDAC WT. Indeed, embryos injected in either right blasomere with the HDAC DN, and embryos injected with HDAC WT on the left side, exhibited heterotaxia at stage 45 (Figure 1B). Embryos injected on the dorsal left side with HDAC DN or HDAC WT did not present significant levels of heterotaxia when compared to un-injected controls.

These data are consistent with a role for histone acetylation during LR development and demonstrate a LR-differential dependence on endogenous HDAC activity. Importantly, these data are not consistent with involvement of the HDAC pathway in cilia-driven events in the gastrocoel roof plate (GRP). The ciliated GRP cells derive from the dorsal precursors [48], whereas our results show effects on ventral blastomere descendants (which do not contribute to the ciliated organ); moreover, it is now known that only the left side of the nodal flow is important for asymmetry at neurula stages [49], while our data reveal effects on the descendants of right-side blastomeres.

As *Nr1 *is the most upstream gene known in the asymmetric cascade of the frog embryo (expressed only on the left side), we processed injected embryos at the stage 21 with a probe for *Xenopus Nr1 *1 (*Xnr-1*) in order to determine whether the pattern of *Xnr-1 *expression was disrupted when early HDAC activity was perturbed. Indeed, embryos injected on the right side with HDAC DN or on the left side with HDAC WT exhibited a loss of the normal asymmetry of *Xnr-1 *expression (HDAC DN on the right: 50% absence of *Xnr-1*; HDAC WT on the left: 26% bilateral expression of *Xnr-1 *with only 5% of control embryos not showing *Xnr-1 *expression).

These results support a role for HDAC activity in LR establishment and suggest that the epigenetic status of the chromatin may play a key role in determining the normally left-sided expression of *Xnr-1*.

### Only early *Xenopus *HDAC blockade affects the Left Right Establishment

We next characterized the timing of epigenetic controls of *Xnr-1 *expression, probing the function of HDAC at the early developmental stages when 5HT signaling takes place [15]. We used a HDAC blocker, Sodium Butyrate (NaB), capitalizing on the ability to use pharmacological reagents at different developmental stages (mRNA injections do not offer temporal control). *Xenopus *HDAC class I activity present in early embryos has already been shown to be sensitive to HDAC blockers [30].

**Figure 2:**
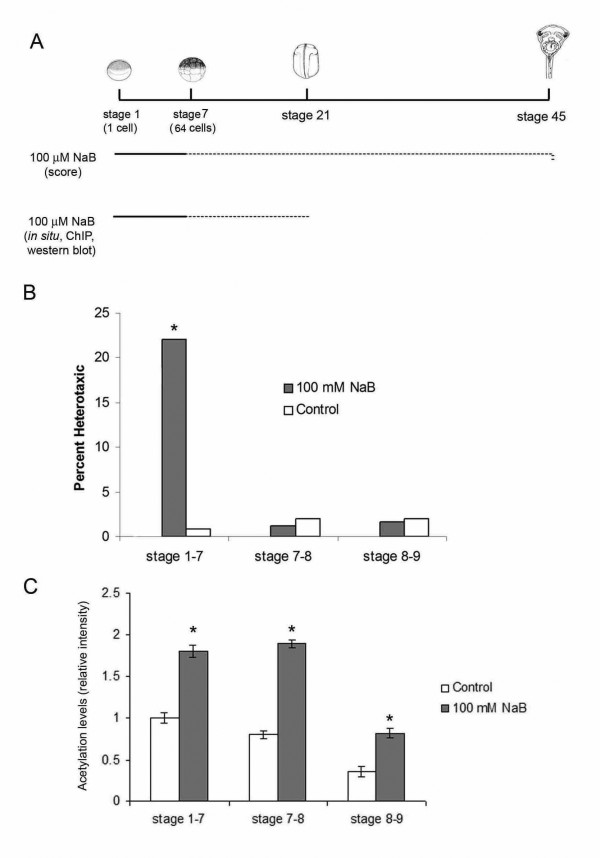
**HDACs exert stage specific effect on left-right patterning**. (A) Schematic showing the experimental procedure for NaB treatments. Embryos were exposed to 100 mM NaB from stage 1 to 7 (solid black line) and the drug was washed out (dashed black line). Embryos were collected at stage 7 for anti-acetyl H4 western blotting or were allowed to develop in 0.1X MMR until stage 45 to score organ placement or until stage 21 for ChIP experiments. (B) 100 mM NaB treatment exerts stage specific effects on left-right development. Embryos that were exposed to NaB between stage 1-7 presented significant levels of heterotaxia (100 mM NaB: 22.5%, *p *< 0.001). NaB exposure after stage 7 does not affect left-right development (stage 7-8 and stage 8-9). (C) NaB treatment led to a significant increase in the H4 acetylation levels. Embryos exposed to NaB from stage 1 to 7 presented 1.8 fold increase, stage 7-8 presented 2.3 fold increase and stage 8-9 presented 2.3 fold increase on the levels of acetylated histone H4 when compared to controls. (* *p *< 0.05).

Embryos at different developmental stages were separated into control groups and experimental groups and the latter were exposed to 100 mM NaB (Figure 2A). Incubation of embryos with NaB induced heterotaxia when exposures occurred between stage 1 and 7 (Figure 2B). Exposure to NaB at any stage after stage 7 did not induce heterotaxia (Figure 2B and Table 1), confirming that the LR-relevant functions of HDAC took place at cleavage stages (under zygotic transcriptional silence and long prior to cilia-mediated flow).

**Table 1:** NaB treatment affects *Xenopus *left-right development

100 mM NaB	stage 1-7	stage 7-8	stage 8-9	stage 13-19	stage 13-15	stage 15-19	stage 19-22
Percent Heterotaxic	22.5%	2%	1.2%	0	0	0	0

Non-scorable	0	0	0	100%	100%	100%	100%

n	145	168	176	150	120	145	160

*p value*	*P <*0.001	*p = *0.6	*p = *0.8				

NaB treatment affects *Xenopus *left-right patterning.

Embryos were exposed to 100 mM NaB during different stages of development, the drug was washed out and the embryos were allowed to develop until at stage 45 for organ placement score. NaB treatment induced significant levels of heterotaxia if embryos were exposed to the drug from stage 1 to 7. Embryos exposed to the drug during *Xenopus *ciliogenesis (stage 13-19; 13-15; 15-19) or during *Nr-1 *asymmetric expression in the lateral mesoderm plate (stage 19-22) presented significant malformations and thus could not be used for laterality assessment.

We also processed NaB treated embryos for *Xnr-1 in situ *hybridization in order to correlate levels of heterotaxia with misexpression of *Xnr-1*. Interestingly, a high percentage of embryos exposed to NaB lacked *Xnr-1 *(64% of *Xnr-1 *absence; Control: 5%). This reproduced the loss of *Xnr-1 *expression resulting from the HDAC DN mRNA microinjection (that was absence of *Xnr-1 *expression).

Thus, these results indicate that the time window most sensitive to blockage of HDAC overlaps with the developmental window in which the 5HT pathway was shown to be active [15].

### HDAC blockade increases the levels of acetylated histone H4 in the early embryo

Given that the normal function of HDAC is associated with a loss of immunoreactivity related to acetylated nuclear H4 [36], and as histone H4 is found in the *Xenopus *egg and early embryos [50,51], we investigated the impact of NaB-induced HDAC blockade on global H4 acetylation levels. Whole protein lysates from embryos exposed to NaB were analyzed by western blotting with a specific antibody against acetylated histone H4. This analysis showed that the histone H4 acetylation levels were markedly increased by 100 mM NaB treatment compared with control groups (Figure 2C). There was a 1.8-fold increase in acetylation levels in embryos exposed from stage 1-7, and a 2.3-fold increase in embryos exposed from stage 7 to stage 8 or from stage 8 to stage 9 (Figure 2C).

These results are consistent with HDAC functioning in the control of histones' acetylation levels during its regulation of LR asymmetry establishment.

### The *Xnr-1 *intronic region that contains the asymmetric element is targeted by the epigenetic machinery

**Figure 3:**
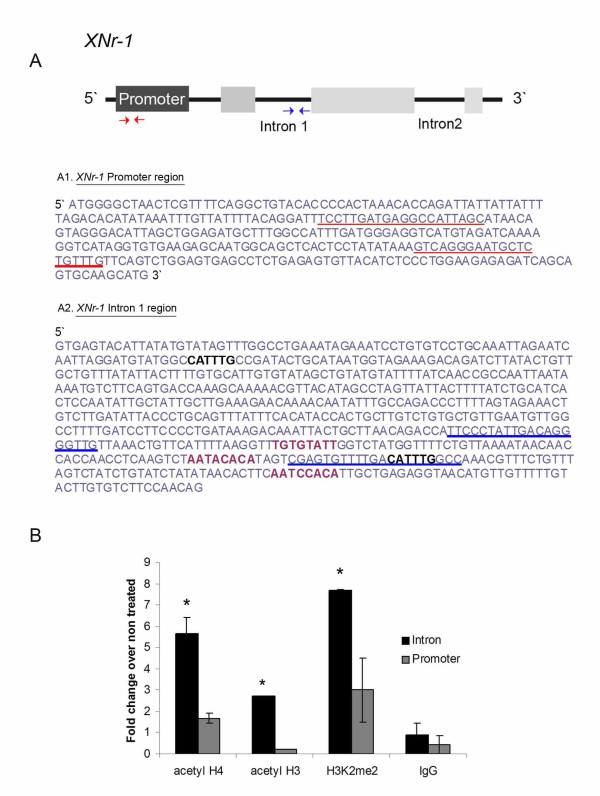
**HDAC inhibition leads to increased levels of acetylated histones and H3K4me2 on the *XNr-1 gene***. (A) Schematic showing the structure of the *Xenopus Nr-1 *gene. Light gray boxes represent the protein-coding and the dark gray box represents the promoter region (adapted from [53]). Intronic regions 1 and 2 are indicated. Red and blue arrows represent the primer set used for qPCR reaction for the promoter and intronic region, respectively. (A1) The red lines indicate the sequence of the *XNr-1 *promoter region used to design the primer set for qPCR reaction. (A2) The regions underlined show the sequence used for primer set design. In purple are highlighted the FAST binding domains as in [53] and the black CATTTG indicates two putative Mad binding sites. (B) Chromatin isolated from embryos exposed to NaB from stage 1-7 and allowed to develop until stage 21 in 0.1X MMR was used for ChIP with anti-acetyl H4, anti-acetyl H3, anti-H3K4me2 and rabbit IgG (Control) followed by qPCR analysis against the promoter region (gray bar) and intronic region (black bar) of *XNr-1*. The levels of acetylated H4 and H3 and H3K4me2 were increased only on the intronic region (black bars).

Our data indicate that very early epigenetic modifications control expression of *Xnr-1 *at a much later developmental stage. Thus, we tested the prediction that this epigenetic modification was made directly on the *Xnr-1 *gene. The *Xnr-1 *genomic region is composed of 2 main regulatory elements (RE). The RE on the promoter region (PRE) is located 230 bp from the start codon [52]. The second RE is the ASE (asymmetric element) in the intron 1 of the *Xnr-1 *gene. It is known to drive the asymmetric expression of *Xnr-1 *in the Lateral Plate Mesoderm (LPM) [53] (Figure 3A).

Because early HDAC activity is necessary for the expression of *Xnr-1 *at stage 21, we asked whether the deposition of epigenetic markers due to early HDAC blockade could cause *Xnr-1's *repression later in development. More specifically, we also sought to map which region of the *Xnr-1 *gene (the promoter or the intronic region that contains the asymmetric element) was a target for the HDAC machinery, and link the deposition of epigenetic markers to the repressed state of *Xnr-1 *induced by NaB treatment. We performed a Chromatin Immunoprecipitation (ChIP) assay with chromatin from embryos at stage 21 that had been exposed to NaB early during development. We focused on three different markers: acetylated histones H4 and H3 (as HDAC inhibition leads to increased levels of histone acetylation), and H3K4me2, because of the existence of crosstalk between histones' modifications [54] and the fact that H3K4me2 modification is also associated with a repressed state of chromatin [55,56]. The H3H4me2 marker was particularly interesting because it has been associated with the epigenetic memory that precedes transcriptional control by several hours [57], thus being an attractive candidate to transmit an epigenetic memory from early to late developmental stages in absence of transcription.

Embryos were exposed to 100 mM NaB from stage 1 to 7 and allowed to develop in plain MMR medium until stage 21. At this stage the chromatin was isolated as described [40,41]. The ChIP experiment followed by qPCR analysis revealed that the level of acetylation of histones H3 and H4 and the levels of H3K4me2 in embryos exposed to NaB early during development presented increased levels only on the intronic region (Figure 3B), suggesting that higher levels of acetylated H3 due to HDAC treatment led to deposition of di-methylated H3 on the intronic region.

Taken together, these results indicate that NaB treatment during early cleavage stages led to a hyperacetylated status of the chromatin and perturbed LR-relevant gene transcription occurring at late developmental stages.

### A high-throughput proteomic analysis identified the HDAC partner Mad3 as a new component in 5HT signaling pathway

Our evidence for epigenetic mechanisms operating at cleavage stages controlling much later transcriptional readouts in LR patterning led us to investigate how HDAC activity could be modulated during early development. Our model predicts such controls, because the HDAC mRNA is expressed symmetrically; thus, an asymmetric signal is needed to confer the observed consistent difference in HDAC activity on the L and R sides. Because rightward redistribution of maternal serotonin (5HT) during cleavage stages has been shown to be necessary for the establishment of LR asymmetry [14,16], we sought mechanisms by which 5HT could couple to the epigenetic machinery.

We performed a high-throughput proteomic assay to identify novel 5HT binding proteins (SBP) present in the cytoplasmic fraction that may interact with known epigenetic machinery. A resin conjugated with 5HT was used for affinity capture analysis from whole protein lysate of *Xenopus laevis *embryos at the 1 cell stage. Two aliquots of 5HT-conjugated resin were loaded with either frog embryo lysate plus 20 mM 5HT (negative control) or frog embryo lysate (test). The eluate from both experiments was subjected to a 10% SDS-PAGE and, after proper sample preparation and trypsin in-solution digestion, the peptide mixtures were analyzed by LC/MS-MS.

The raw data collected from the ion trap mass spectrometer was analyzed for protein identification using a database for *Xenopus laevis*. The screen identified 11 proteins as possible 5HT interactors. Because we were interested in mechanisms by which intracellular 5HT binding proteins could couple to the cytoplasmic epigenetic machinery (as 5HT is delivered inside the cell, via gap junctions [16]), we focused on candidate proteins with known interactions with HDAC proteins and/or DNA-binding domains. We did not recover the classical 5HT transmembrane receptors in this screen, since we focused our analysis on the cytoplasmic (not the plasma membrane) protein fraction. Indeed, our screen successfully identified 10 candidates with characteristics of cytoplasmic proteins and 1 candidate having a DNA binding domain. This DNA binding-protein was identified as the Max-interacting transcriptional repressor Mad3. Mad3 is involved in the control of the epigenetic state of cells, since it can antagonize transcription by recruiting co-repressor complexes that contain HDAC I [58]. Indeed, the repressive activity of Mad proteins has been shown to be due to HDAC activity and inhibited by HDAC blockers [59]. Mad proteins also interact with Max, and this complex has been implicated in different aspects of cell biology and cancer transformation [60].

### Mad3 protein and mRNA are present in the early embryo

**Figure 4:**
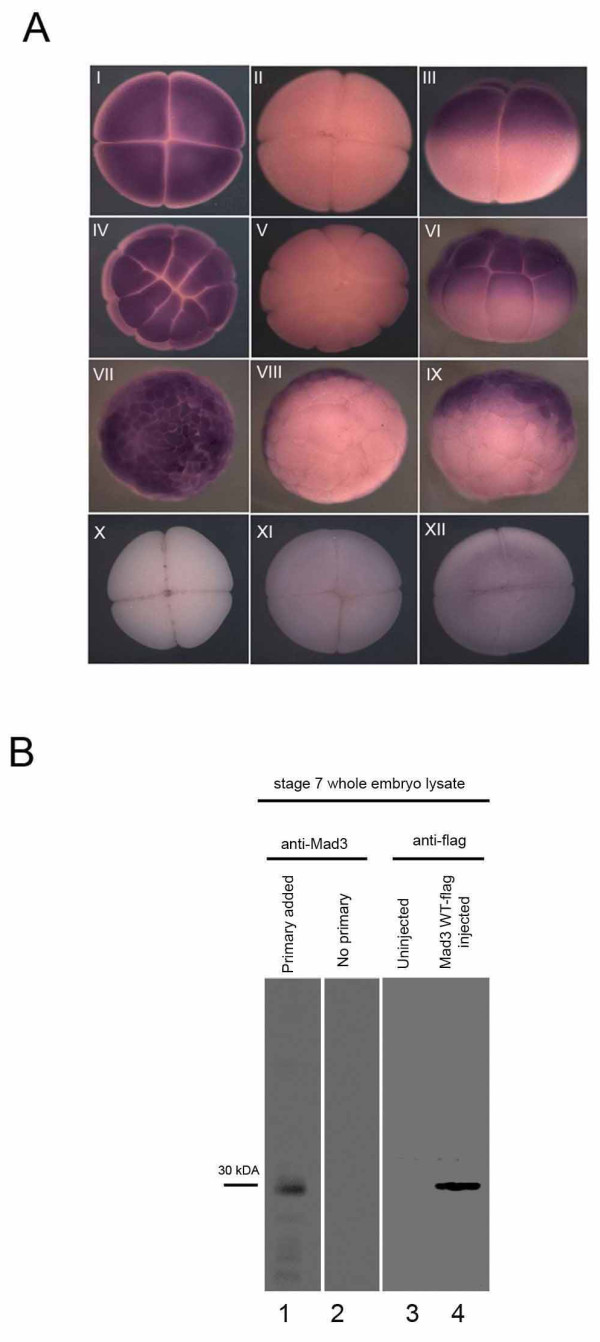
***Xenopus *Mad3 mRNA and protein are present in early embryos**. (A) Mad3 mRNA is expressed by animal cells in a symmetric pattern at the stage 3 (4 cells) (I,II,III), at the stage 5 (16 cells) (IV,V,VI) and at the stage 7 (64 cells) (VII,VIII,IX). Figure X: *in situ *reaction control, animal view (no secondary antibody added to the reaction); XI and XII: no probe added to the reaction, animal and vegetal view, respectively. (B) Immunoblotting using stage 7 (64 cells) whole embryo lysate with an anti-Mad3 antibody. Lane 1: Whole lysate from stage 7 embryos. Anti-Mad3 recognizes a band running at 30 kDa. Lane 2: Immunoblotting control with no primary antibody added to the membrane. Lanes 3 and 4: Whole protein lysate from non injected (lane 3) or Mad3WT-flag injected embryos (lane 4) were used to detect exogenous Mad3.

A role for Mad3 as a new player in the 5HT signaling pathway during LR patterning requires that Mad3 protein be already present in early embryos. Indeed, Mad3 mRNA is present in the blastomeres at the 4-cell stage by *in situ *hybridization (Figure 4A). By the 32-64 cell stage (stage 6-7), Mad3 mRNA is found only in the animal cells.

To validate the presence of Mad3 protein in early embryos, as detected on the ion trap mass spectrometer, we performed two different sets of analysis. In the first strategy we used a monoclonal anti-Mad3 antibody to detect the endogenous Mad3 protein in embryos at stage 7 (Figure 4B, lane 1). To confirm the specificity of this anti-Mad3 antibody we injected embryos with the *Xenopus *Mad3 protein fused to the flag epitope at the 1 cell stage. The lysate was collected at the stage 7 and used for immunoblotting to detect the exogenous *Xenopus *Mad3. Indeed, the injected Mad3-flag protein was detected migrating at 30 kDa, the expected molecular size for the *Xenopus *Mad3 protein (Figure 4, lane 4). Taken together these results confirm that Mad3 is present as a maternal mRNA and protein at the early cleavage stages and thus is potentially available for interaction with 5HT and/or HDAC.

### Mad3 is a new player during *Xenopus *LR patterning

To examine a role for Mad3 during LR patterning, we utilized 2 different constructs encoding loss-of-function and gain-of-function reagents for Mad3: Mad3Vp16 (a constitutively active form of Mad3) and EngMad3 (a constitutively repressive form of Mad3) [61].

**Figure 5:**
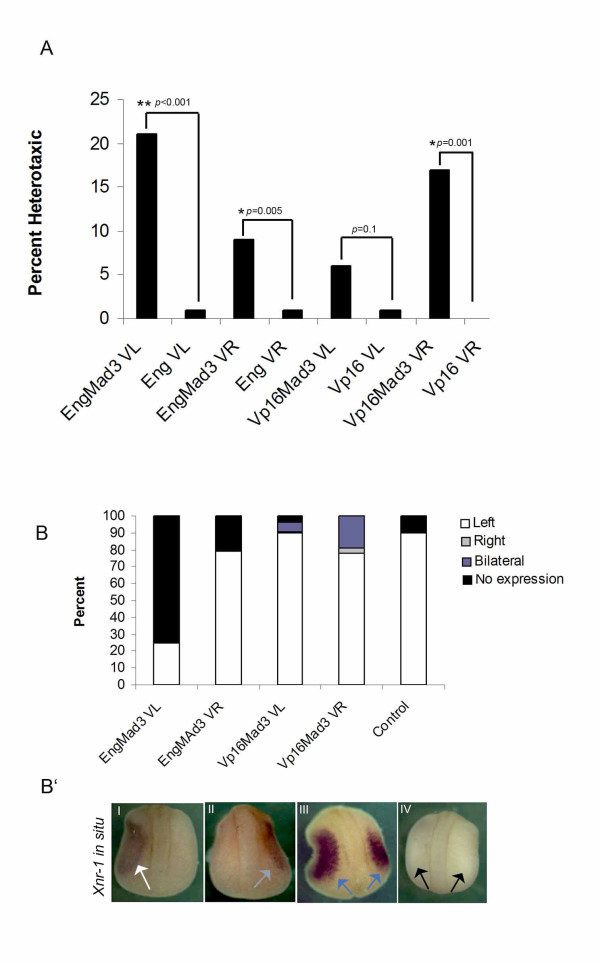
**Mad3 is a new left-right determinant**. (A) Single ventral left or ventral right blastomeres were injected with EngMad3 or Vp16Mad3 constructs and organ placement was scored at stage 45. Significant levels of heterotaxia were observed when embryos were injected with EngMad3 (left: 21%, n = 96, *p *< 0.001; right: 12%, n = 91, *p *= 0.005; control: Eng) or with Vp16Mad3 on the right side (17% n = 90, *p *< 0.001; 6% n = 127, *p *= 0.1, control: Vp16). (B) Embryos injected with EngMad3 or Vp16Mad3 were processed for *in situ *hybridization at stage 21 with a *XNr-1 *probe. *XNr-1 *misexpression was correlated with Vp16Mad3 injection (right injected embryos: 19% bilateral expression, n = 117; left injected: 5% bilateral expression, n = 105) and EngMad3 (left injected: 75% no expression, n = 74; right injected: 21% no expression, n = 93) if compared to control embryos (uninjected, 10% no expression, n = 103). (B') *XNr-1 *expression pattern characterized in EngMad3 or Vp16Mad3 injected embryos. I- Left expression indicated by the white arrow; II-right expression indicated by the gray arrow; III- Bilateral *XNr-1 *expression indicated by the two blue arrows; IV- Absence of *XNr-1 *expression as indicated by the two black arrows.

To probe the requirement of Mad3 between the left and right side of the embryo, the single dorsal or ventral, left or right blastomeres at the stage 3 (4 cells) were injected with mRNA encoding EngMad3 or Vp16Mad3 along with the lineage tracer *lacZ *mRNA. The embryos were then scored for organ placement at stage 45. Whereas dorsal injections led to considerable toxicity, ventral left or right expression of the EngMad3 (repressive) construct induced heterotaxia at a statistically significant level when compared to control Eng-expressing embryos (Figure 5A). Conversely, embryos injected with Vp16Mad3 in the right ventral blastomere presented significant heterotaxia when compared with Vp16 control injected embryos (Figure 5A).

We also analyzed injected embryos with EngMad3 or Vp16Mad3 by *in situ *hybridization with a *Xnr-1 *probe. Indeed, embryos expressing the repressive EngMad3 construct on the left or right side showed *Xnr-1 *absence, and embryos injected with the activating Vp16Mad3 on the right showed a consistent level of bilateral *Xnr-1 *expression (Figure 5B).

Thus, Mad3 gain-of-function on left (EngMad3) and a Mad3 loss-of-function on the right (Vp16Mad3) can both control *Xnr-1's *transcriptional status. Taken together, these results support Mad3 as a new LR determinant, and are consistent with a role for Mad3 as a modulator of *Xnr-1 *expression.

### The Mad3 biological activity during LR establishment is dependent on 5HT

**Figure 6:**
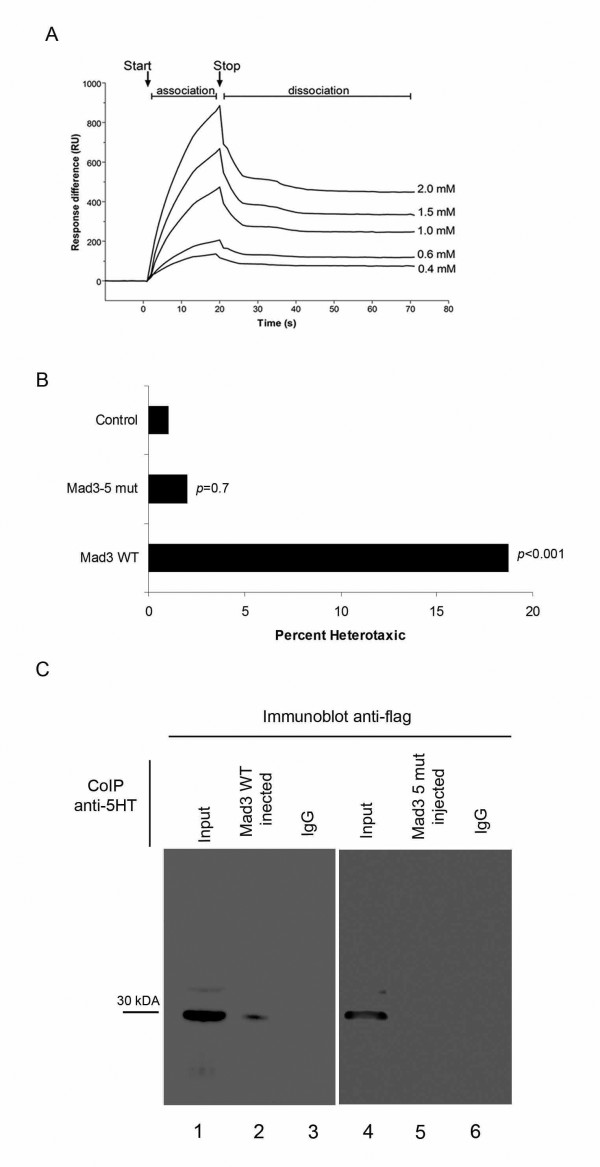
**Mad3's biological activity depends on 5HT**. (A) Sensorgram of serotonin binding to GST-Mad3. Data represent the difference between the sample cell (GST-Mad3) and the reference cell (GST). A blank run (buffer only) has been subtracted from all curves. Four different analyte (5HT) concentrations (2, 1.5, 1, 0.8 and 0.4 mM were injected). Injection start and stop are indicated by arrows. The data shown are representative of 3 independent experiments. (B) Embryos were injected at stage 1 with Mad3 WT or Mad3-5mut and scored for left-right phenotype at stage 45. Embryos injected with Mad3 WT presented significant levels of heterotaxia (17%, n = 52, *p *= 0.001) whereas Mad3-5mut injected embryos presented only 2% of heterotaxia (n = 104, *p *= 0.7) when compared to control group. (C) Co-Immunoprecipitation assay. *Xenopus *embryos at the 1 cell stage were injected with Mad3 WT-flag (lanes 1,2,3) or Mad3-5mut-flag (lanes 4,5,6) and collected at the stage 7 (64 cells). The whole protein lysates were incubated with anti-5HT antibody followed by protein-A agarose. Lane 1: 10% Input; Lane 2: whole lysate from Mad3 WT-flag injected embryos plus anti-5HT; Lane 3: negative control (whole lysate from Mad3 WT injected embryos plus rabbit IgG); Lane 4: 10% input; Lane 5: whole lysate from Mad3-5mut-flag injected embryos plus anti-5HTLane 6: negative control (whole lysate from Mad3-5mut-flag plus rabbit IgG).

Next we tested whether Mad3's biological activity is dependent on 5HT. The first strategy was to carry out a binding assay using surface plasmon resonance (SPR) [62]. Recombinant Mad3 protein expressed as a fusion with glutathione S-transferase (Mad3-GST) was immobilized in a Biacore chip and a 5HT solution was then passed over the surface. To subtract possible non-specific binding, we used a reference surface in the same chip where GST alone was immobilized. 5HT showed a high association rate and a low dissociation rate (Figure 6A). Four different concentrations were examined, ranging from 400 μM to 2 mM. Using a 1:1 Langmuir fitting algorithm, we calculated the equilibrium dissociation constant to be in the range of 6.7 to 26 μM (95% confidence). Based on the 5HT levels found in early embryos, 3.11 pmol/egg [15], we measured embryo diameters in order to calculated the endogenous concentration of 5HT. Considering the whole embryo as a sphere (diameter = 1mm), 5HT concentration can then be calculated as 5.7 μM, which is very close to the Kd values determined *in vitro*. It should be noted that 5HT is not homogeneously distributed in the whole embryo but concentrated in the right blastomeres descendants at the stage 7 [15]; therefore, the local concentrations could be even higher than the ones calculated here, well above the determined dissociation constant. Hence, these *in vitro *data does suggest that 5HT and Mad3 form a complex *in vivo*.

The second strategy was to generate a Mad3 mutant lacking 5HT-binding sites. To generate this Mad3 mutant first we mapped the putative 5HT binding sites on Mad3 by modeling the 3-dimensional structure for Mad3 using the crystal structure of a lipocalin AM182, a well characterized 5HT-binding protein, complexed with 5HT [43]. We chose lipocalin because among all known 5HT binding proteins, including class-A GPCRs (G protein-coupled receptors), a conserved salt bridge interaction exists between the amine group and an aspartic acid or glutamic acid residue on the protein [45,63-65]. In lipocalin AM182, this conserved salt bridge is formed between Asp106 and the amine group of 5HT. The structural equivalent aspartic acid in Mad3 is Asp163. Thus the potential binding site for 5HT in Mad3 was derived based on the corresponding binding site residues from lipocalin structure.

To gain insight into the physiological relevance of these putative 5HT binding sites on Mad3, we performed functional experiments with a Mad3 mutant generated based on the structural modeling. Based on the docking mode of 5HT in the Mad3 structure, residues D145, D148, D163, Q125 and Q161 are proposed to form important components of the 5HT binding pocket on Mad3 (see Material and Methods). An evaluation of the binding site residues was performed by designing a Mad3-5mut-flag construct harboring mutations on the five amino acids predicted to be involved in the 5HT putative binding site (Q125E, Q161E, D145N, D148N and D163N). Embryos injected with Mad3-WT-flag at the 1 cell stage developed significant levels of randomization of the heart, gut and gall bladder (heterotaxia) in the absence of other defects and with normal dorsoanterior development at stage 45; in contrast, the Mad3-5mut did not induce this phenotype (Figure 6C, Mad3 WT 17%, *p *< 0.001, n = 93; Mad3-5 mut 2%, *p *= 0.7, n = 90).

To confirm that the lack of biological activity presented by the Mad3-5mut-flag was due to the abrogation of the 5HT putative binding site, we performed a Co-IP assay with a 5HT antibody. Because 5HT is evenly distributed in embryos from stage 1 (1 cell) through stage 5 (16 cells), we injected embryos at the 1 cell stage to probe Mad's ability to bind to any available 5HT present in the embryo, and in turn, to test if this ability would be lost in Mad3-5mut injected embryos. Embryos were then injected at the 1-cell stage with Mad3WT-flag or Mad3-5mut-flag and whole embryo lysate was prepared at the stage 7 (64 cells) and incubated with ati-5HT followed by immunoblotting with anti-flag. As predicted, the Mad3-5mut-flag protein was not found in complex with 5HT (Figure 6C, lanes 4,5,6) when compared to the Co-IP performed with Mad3 WT-flag injected embryos (Figure 6, lanes 1,2,3). These results confirm the importance of these 5 residues for the Mad3 biological activity during laterality establishment due to 5HT. We conclude that serotonin binding is a key component of Mad3's ability to functionally participate in LR patterning.

## Discussion

### Chromatin structure and epigenetic state as a new source of asymmetric information

The importance of chromatin remodeling for cancer and developmental biology is increasingly appreciated [66]. The epigenetic state of the cell can be characterized by modifications on histone proteins such as acetylation, methylation, phosphorylation and ubiquitination [67]. One important epigenetic state is characterized by the levels of histone tail acetylation that are tightly controlled by HDACs [68].

Previous work has shown that the *Xenopus *HDAC protein is found at high concentrations in the oocyte nucleus and its levels remain constant through oocyte maturation, fertilization, and early cleavage stages but decreases after the blastula stage [29]. Measurements of *Xenopus *HDAC activity indicate that it also remains constant from oogenesis until early embryogenesis and that it is sensitive to HDAC blockers [30], which is consistent with the data we obtained on its role in the early LR pathway. In addition, our study indicates that although HDAC mRNA is expressed in all animal blastomeres, HDAC activity on the right side is important for LR, as HDAC DN injections on the right side led to heterotaxia that was correlated with absence of *Xnr-1 *expression. In addition, NaB treatment shows that the critical HDAC activity for LR development overlaps with 5HT signaling (right blastomeres) during cleavage stages and takes place before cilia flow (Figure 2). The results of the molecular-genetic loss-of-function (dominant negative construct misexpression) confirm the targeting of the pharmacological reagent (NaB), while the latter offers the opportunity of temporally-limited exposure.

Our results using the ChIP and qPCR analyses indicated that HDAC inhibition led to accumulation of histones H3 and H4 and the epigenetic marker H3K4m2 in the intronic region of *Xnr-1*. This region is particularly critical for *Xnr-1 *asymmetric expression and we propose that HDAC activity targets this region of the *Xnr-1 *gene. Although the H3K4me2 marker has been associated with transcriptional activation when it occupies promoter regions [69], it was also shown to be associated with repression [55]. Interestingly, H3K4me2-enriched regions do not correlate with transcription start sites and have been proposed to control chromatin states over regulatory regions [70]. This feature is particularly interesting as H3K4me2 was found enriched only on the intronic region of *Xnr-1 *that contains the asymmetric regulatory element. One hypothesis is that, after drug removal, the H3K4me2 marker remained stably tethered on the *Xnr-1 *intronic region and led to its repression due to the recruitment of zygotic HDACs during late developmental stages.

### The function of Mad3 in the LR establishment

In agreement with our result indicating that the HDAC block leads to an increase in histone H4 and H3 acetylation levels, Mad signaling was shown to be important in the control of global levels of chromatin structure also characterized by acetylation of heterochromatin regions. Indeed, Mad proteins have been shown to coordinate chromatin modifications resulting in a significant decrease in acetylated histones H3 and H4 in cell culture [71].

**Figure 7:**
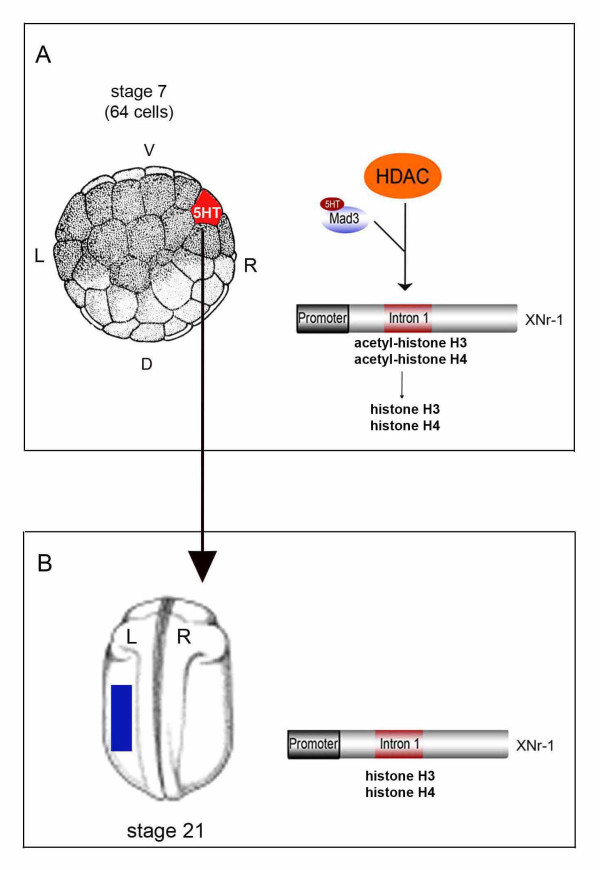
**HDAC activity is required during LR development**. (A) In this study, we found that early HDAC activity between stages 1-7 is important to proper set the pattern of expression of *XNr-1 *at stage 21. In addition, we also show that HDAC activity and Mad3 are important on the right side of the embryo to set *XNr-*1's expression and that Mad's biological activity is dependent on 5HT. Despite HDAC and Mad3 mRNAs present a symmetric pattern of expression, one theory is that because 5HT is asymmetrically distributed in the right blastomeres at stage 7 (red), 5HT binding on Mad3 (blue) may recruit HDAC activity (orange) to the intronic region of *XNr-1 *on the right of the embryo. HDAC activity, at early developmental stages, may be important to decrease the levels of acetylation of histone H3 and H4 on the intronic region of *Nr1*. (B) At stage 21, low levels of acetylation on histones (H3 and H4), set by early HDAC activity, would contribute to repress the expression of *Nr1*, that will be expressed only on the left side of the embryo (blue box). V-ventral; D-dorsal; R-right; L-left.

A key feature of 5HT signaling during *Xenopus *LR development is that it functions during cleavage stages, prior to mid-blastula transition (MBT), and thus largely under zygotic transcriptional silence. It was previously shown that early 5HT signaling is important to the correct placement of *Xnr-1 *expression on the left side of embryo [15]. Indeed, constitutive repressive form of Mad3 protein (EngMad3) can induce heterotaxia and blocks *Xnr-1 *expression on the left side (Figure 5), which indicates its endogenous role as a repressor of *Xnr-1*. On the other hand, Vp16Mad3 injections could relieve the repressive state of *Xnr-1 *on the right side leading to its ectopic expression at stage 21 (Figure 5). In contrast to transcriptional control of *Xnr-1*, we propose that the EngMad3 phenotype is due to a constitutive recruitment of repressive elements of the cell machinery that can lead to a repressive state of the chromatin that can be maintained throughout development. Conversely, the Vp16Mad3 phenotype is compatible with an open chromatin structure leading to the ectopic induction of *Xnr-1*. Interestingly, injections with the HDAC DN and Vp16Mad3 on the right and HDAC WT and EngMad3 on the left gave rise to consistent heterotaxia, indicating that Mad3 and HDAC functions during LR establishment take place in the same subset of blastomeres and may converge on *Nr1*. This hypothesis is corroborated by the presence of 2 putative Mad binding sites CANNTG [72] in the intronic region of *Nr1 *(Figure 3A2). Interestingly, the second site is placed in the region that contains the FAST binding sites that are important to proper control the asymmetric expression of *Nr1 *in the left side of the embryo [53]. This interesting feature suggests that Mad protein could bind to this region of the *Nr1 *gene. This analysis, along with the established status of Mad3 as a very well-characterized partner for HDACs, indicates that 5HT/Mad3 signaling could couple to repressive elements belonging to the epigenetic machinery of the early embryo. In addition, HDAC and Mad3 mRNA symmetric expression patterns argue in favor of a symmetric distribution for both proteins. In this context, we hypothesize that 5HT binding on Mad3 would be an asymmetric signal important to confer specificity for HDAC activity in the context of LR development, decreasing the levels of histone acetylation on the *Nr1's *intronic region (Figure 7).

Indeed, a recent study has demonstrated an antidepressant-like effect for HDACs inhibitors similar to the effects of fluoxetine, an effective inhibitor for 5HT re-uptake, [73] that randomizes LR asymmetry [16]. These data suggest a synergistic role for 5HT and HDAC pathways, indicating they may be acting in the same pathway and reinforcing our model that brings together 5HT and epigenetic machinery.

### How is the early LR signaling pathway transduced into a stable gene expression pattern at a late developmental stage?

In *Xenopus *and many invertebrates, consistent asymmetry is determined by very early biophysical and physiological events taking place long before asymmetric gene expression and ciliary flow [10,11]. While these early mechanisms are mapped onto different embryonic architectures in a variety of ways throughout phyla [74,75], left-sided *Xnr-1 *expression is a well-conserved regulator of the *situs *of the heart and visceral organs [76]. Our data on epigenetic modulation provide the first detailed glimpse into the molecular events that allow physiological events during very early stages to be solidified into cascades of gene expression.

Analysis of the mouse and *Xenopus Nr-1 *gene has revealed a regulatory sequence in the coding region that is crucial for the asymmetric expression at the LPM and that is targeted by the complex FAST/SMADS [53,77]. This asymmetric enhancer (ASE) sequence is present in the intronic region [53]. The transcription factor FAST-1 mediates TGFβ signaling, and, together with SMAD-2 and -4, has been shown to be necessary to trigger *Xnr-1 *asymmetric expression by binding to the *Xnr-1 *ASE in the LPM [77]. However, all signaling molecules that play a role in the asymmetric expression of *Nr-1 *characterized so far are symmetrically expressed in the LPM, including the immediate upstream player FAST-1 [77]. Thus, it becomes crucial to understand how upstream symmetric events taking place at the cellular levels result in reliably asymmetric *Nr-1 *expression. In addition, it is known that the right side of the embryo has an intrinsic ability to express *Xnr-1*, indicating that the cells on the right side have all machinery needed to express *Xnr-1 *but are normally repressed from doing so [78].

Our results show that the *Xnr-1 *intronic region contains high levels of acetylated histone H3 and H4 and H3K4me2 after NaB treatment (Figure 3) and this correlates with absence of *Xnr-1 *expression. Although the biological significance in terms of transcriptional outcomes due to H3K4me2 is still under debate, it is becoming clear that this epigenetic marker may prevent aberrant gene expression or modulate transcriptional outcomes [55]. In the context of our results, a possible interpretation is that H3K4me2 could work as a repressive marker facilitating the efficiency of inhibition by Lefty. In normal embryos these results suggest that HDAC could target the *Xnr-1 *intronic region (ASE) early during development leading to a decrease in the levels of acetylated histones H3 and as a consequence preventing H2K4me2 from being deposited in this region, making this region accessible to FAST related proteins. By the time of *Xnr-1 *expression initiation, the absence of H3K4me2 would increase the efficiency of activation of *Xnr-1 *expression, which will not result in significant noise, leading to stable expression of *Xnr-1*.

Future experiments addressing the balance in acetylation and methylation levels of histones between left and right sides of the embryo will be necessary to understand how the epigenetic machinery controls different elements during LR determination besides *Nr1*. For instance, the investigation of epigenetic modifications on other left-right genes, such as *Lefty *and *Pitx *will be important to understand how global HDAC activity blockade changes the chromatin status and how these changes are transduced into different states of LR genes' activity. For example, *Lefty *is an inhibitor of *Nr1 *and any epigenetic change on *Lefty *due to HDAC blockade may also affect *Nr1 *expression, providing a possible explanation on the absence of *XNr-1 *expression when HDAC activity is blocked. This hypothesis is supported by the feed-forward and feedback loop between *Nr1 *and *Lefty *that is important to exclude Nodal from being expressed on the right side of the embryo. Consistent with this rationale, our data implicate HDAC activity as important to set *Nr1 *expression but also suggest that HDAC activity may target *Lefty*, leading to its ectopic expression on the left side that ultimately will repress *Nr1 *expression. For this reason, a comprehensive understanding of the epigenetic regulation of the key asymmetric genes, and the upstream components linking the sidedness of transcription to early physiological gradients, will be a crucial aspect of fleshing out a most fascinating aspect of left-right patterning.

## Conclusions

HDAC activity is a new LR determinant controlling the transcription of the *Xnr-1 *gene. Molecular-genetic and pharmacological blockade of HDAC activity led to deposition of epigenetic markers on the *Xnr-1 *gene that was correlated with misexpression of *Xnr-1 *and organ heterotaxia. The known HDAC partner Mad3 is also a new functional LR determinant whose biological activity during LR establishment is dependent on 5HT. Taken together these data suggest a model in which epigenetic machinery transduces early physiological gradients into much later transcriptional effectors during establishment of consistent organ *situs *in vertebrate embryogenesis.

## Authors' contributions

KC designed and carried out the experiments, and wrote the manuscript. BK, CD, and TR conceived and CD and TR carried out the Chromatographic 5HT affinity capture screening. GB and ED designed and GB carried out the Mad3 and 5HT *in vitro *interaction assay (Surface Plasmon Resonance assay). KC and ML interpreted the data and designed experiments. SK carried out the Mad3 modeling and simulation. JML designed and constructed many of the plasmids. ML conceived, designed, and wrote the manuscript. All authors read and approved the final manuscript.
